# Fracture and relaxation in dense cornstarch suspensions

**DOI:** 10.1093/pnasnexus/pgad451

**Published:** 2023-12-22

**Authors:** Paul Lilin, Jean E Elkhoury, Ivo R Peters, Irmgard Bischofberger

**Affiliations:** Department of Mechanical Engineering, Massachusetts Institute of Technology, Cambridge, MA 02139, USA; Schlumberger-Doll Research, Cambridge, MA 02139, USA; Faculty of Engineering and Physical Sciences, University of Southampton, Highfield, Southampton SO17 1BJ, UK; Department of Mechanical Engineering, Massachusetts Institute of Technology, Cambridge, MA 02139, USA

**Keywords:** jamming, shear thickening, fracture, relaxation

## Abstract

Dense suspensions exhibit the remarkable ability to switch dynamically and reversibly from a fluid-like to a solid-like, shear-jammed (SJ) state. Here, we show how this transition has important implications for the propensity for forming fractures. We inject air into bulk dense cornstarch suspensions and visualize the air invasion into the opaque material using time-resolved X-ray radiography. For suspensions with cornstarch mass fractions high enough to exhibit discontinuous shear thickening and shear jamming, we show that air injection leads to fractures in the material. For high mass fractions, these fractures grow quasistatically as rough cavities with fractured interfaces. For lower mass fractions, remarkably, the fractures can relax to smooth bubbles that then rise under buoyancy. We show that the onset of the relaxation occurs as the shear rate induced by the air cavity growth decreases below the critical shear rate denoting the onset of discontinuous shear thickening, which reveals a structural signature of the SJ state.

**Significance Statement**

Shear-thickening suspensions can switch reversibly between a fluid-like and a solid-like jammed state. This phenomenon has practical implications for the behavior of bulk materials ranging from chocolate and cement to ocean floor sediments and magma, which can jam, fracture, and relax back to a flowing state. Much of the research into these phenomena has, however, focused on simplified geometries and on rheology. Here, we employ time-resolved X-ray radiography to probe the fascinating formation of fractures and the subsequent relaxation occurring inside a bulk cornstarch suspension upon air injection. Our findings establish a link between fracturing and shear-jamming in dense suspensions, and encourage further studies addressing relaxation in continuously deforming suspensions.

## Introduction

Dense suspensions are complex fluids that can exhibit discontinuous shear thickening, where the suspension viscosity increases by several orders of magnitude as a critical shear rate is reached (1–8). Dense suspensions can also jam under shear and behave like solids that bear weight and fracture in a variety of dynamic settings including impacts, under extensional flow, or when air is injected in a Hele-Shaw cell filled with the suspension (9–14). The rheology of aqueous cornstarch suspensions has been studied extensively and the material has become the prototypical example of a shear-thickening and shear-jamming fluid (4, 8, 11, 15–18).

The onset of discontinuous shear thickening (DST) occurs as the particle interactions transition from lubricated to frictional contacts. The frictional contacts lower the critical particle volume fraction at which the system jams compared to the stress-free lubricated case (6–8, 19–23). If the stress applied to the suspension is increased further, the suspension can enter a shear-jammed (SJ) state (24–27). Two conditions are thus necessary for the suspension to shear jam: the applied shear rate must reach the critical shear rate denoting the onset of DST, and the applied stress must be larger than the critical stress denoting the onset of SJ. As the mass fraction of particles increases, both the critical shear rate and the critical stress decrease, widening the range of conditions in which shear jamming occurs (6, 26, 28). While the transition from a flowing to a shear-thickening or SJ state has been well studied, including the propagation of a jamming front (10, 26, 29, 30), the relaxation out of a SJ state is just starting to be explored (28, 31–33). In addition, the conditions for fracture of SJ dense cornstarch suspensions are complex, and the critical stresses and strains triggering fracture have been reported to depend on the shear rate and on the geometry under consideration (9, 11, 13).

An insightful geometry for studying complex fluids is that of fluid injection from a nozzle. Air injection into most liquids leads to the formation of smooth bubbles which rise upwards under the effect of buoyancy, adopting various shapes and terminal velocities depending on the liquid rheology (34–40). Conversely, fluid injection into a solid material creates a thin fracture perpendicular to the injection nozzle, as elastic stresses prevent the fracture from widening (41–44). In viscoelastic materials, the shape of the air cavity typically depends on the timescale of air injection compared to an intrinsic timescale in the material (45–48). Whether a material fractures or flows becomes a more difficult question in dense suspensions due to their complex rheology. Understanding the shape and motion of air inside a dense suspension will give insight into air bubble stability in concrete where bubbles are used to increase thermal insulation and thaw resistance (49, 50), might help guard against bubbles rising in a wellbore in offshore oil and gas drilling (37, 51), and will contribute to revealing air dynamics in magma (52, 53).

Here, we probe the formation of fractures and their relaxation in bulk dense cornstarch suspensions. We employ time-resolved X-ray radiography to visualize the response of the suspension to air injection through a nozzle placed at the bottom of the container. Operating within the range of mass fractions where the suspension exhibits DST, we observe stark differences in both the motion and the shape of the air cavity as we vary the cornstarch mass fraction. For the higher range of mass fractions, the suspension fractures upon air injection. The large solid-like fractures expand into cavities with fractured interfaces that do not relax during their growth. The air cavities show no sign of buoyancy in their vertical motion. For the lower range of mass fractions, the suspension also fractures initially, but then the interface relaxes into a smooth bubble whose buoyancy-driven vertical motion can be accounted for by considering the suspension as a viscous Newtonian fluid. We show that the relaxation of the suspension is linked to a decrease in the shear rate applied by the cavity growth, which eventually becomes smaller than the critical shear rate denoting the onset of DST allowing the suspension to relax.

## Results

### Growth of air cavities in dense cornstarch suspensions

To inject air into dense cornstarch suspensions, we pressurize air inside a 19 L gas tank to a gauge pressure $P_{g}$. The gas tank is connected via tubing to a nozzle placed at the bottom of a cubic container filled with a dense cornstarch suspension. When the valve between the gas tank and the nozzle is opened, air is injected into the suspension.

**Fig. 1.**
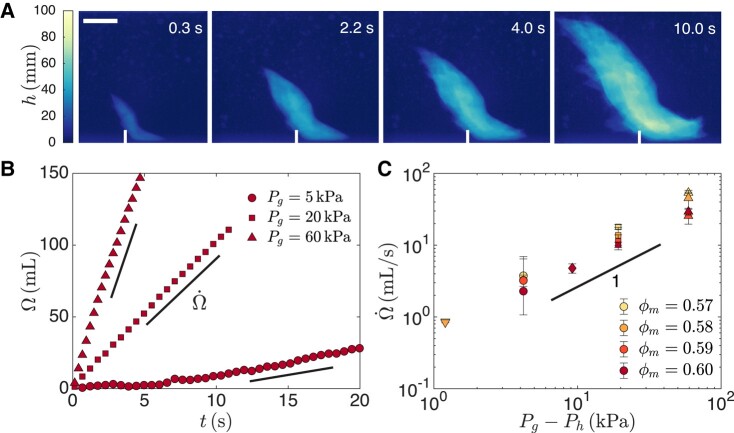
Growth dynamics of air cavities in dense cornstarch suspensions. A) Temporal evolution of the thickness of the air cavity *h* for a cornstarch mass fraction $\phi_{m}=0.60$ and a gauge pressure $P_{g}=20\mathrm{kPa}$. Air is injected into the cornstarch suspension at the bottom of the container through a nozzle. The time is measured from the start of the air cavity growth and the scale bar represents 2 cm. B) Cavity volume *Ω* calculated from the air thickness data versus time for different gauge pressures and $\phi_{m}=0.60$. *Ω* increases linearly with time at a volume rate $\dot{\Omega }$. C) The volume rate $\dot{\Omega }$ increases linearly with the gauge pressure $P_{g}$ and is almost independent of the mass fraction $\phi_{m}$.

We visualize the growth of air cavities in the opaque cornstarch suspension using time-resolved X-ray radiography with a frame rate of 3.73 frames per second. The sample is placed between an X-ray source and a PerkinElmer detector measuring the transmitted X-ray intensity. As air has a lower attenuation coefficient than the cornstarch-water suspension, the air cavity appears as a region of higher transmitted X-ray intensity (54). We convert the transmitted X-ray intensity into an air thickness value *h*, as shown in Fig. 1A for a suspension with cornstarch mass fraction $\phi_{m}=0.60$ and a gauge pressure $P_{g}=20\;\mathrm{kPa}$ (Movie S1).

To probe how fast the cavity grows for a constant tank gauge pressure, we sum the air thickness value h(t) over the entire image and subtract any air volume present in the container before the appearance of the cavity, h(0), to obtain the volume of the cavity Ω(t)=∑(h(t)−h(0)) shown in Fig. 1B. The volume of the cavity exhibits a constant volume rate $\dot{\Omega }$ throughout the cavity growth. The volume rate increases linearly with the difference between the gauge pressure and the hydrostatic pressure at the level of the nozzle, $P_{g}-P_{h}$, but is almost independent of the cornstarch mass fraction $\phi_{m}$, as seen in Fig. 1C. We probe a range of $\phi_{m}$ where the suspension exhibits discontinuous shear thickening, starting from $\phi_{m}=0.57$, the lowest mass fraction for which the cavity growth dynamics is slow enough to be captured in X-ray radiography, to $\phi_{m}=0.60$, the highest mass fraction that can be homogeneously mixed.

The observations that the volume rate is constant and linearly dependent on $P_{g}-P_{h}$ indicate that during the cavity growth, the pressure losses in the tubing connecting the gas tank to the nozzle are large compared to the pressure loss at the air cavity/suspension interface (55) (see Supplementary Text and Fig. S1 for details). As a result, even though we control the tank pressure $P_{g}$, the experiments are effectively flow-rate controlled.

### Role of buoyancy and growth in the rise of the air cavity

**Fig. 2.**
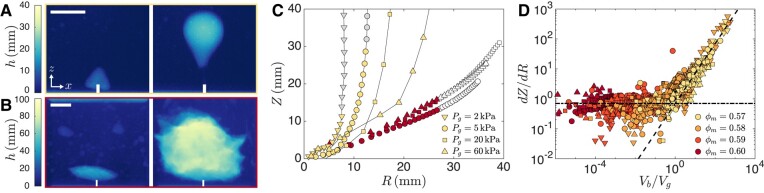
Vertical motion of air cavities in cornstarch suspensions. A) Air thickness images for mass fraction $\phi_{m}=0.57$ and gauge pressure $P_{g}=5\mathrm{kPa}$ at times t=1.3s and 4.8 s. The air cavity rises up and detaches from the nozzle. B) Air thickness images for $\phi_{m}=0.60$ and $P_{g}=5\mathrm{kPa}$ at t=3.8s and 27.4 s. The cavity remains attached to the nozzle and eventually grows close to the walls of the container. The scale bars represent 2 cm. C) Vertical position of the center of the cavity *Z versus* the equivalent cavity radius $R=(3/(4\pi )\Omega {)}^{1/3}$. For $\phi_{m}=0.57$ (light symbols), *Z* increases faster than *R* due to buoyancy acting on the bubble-like cavity. Gray symbols indicate that the cavity is detached from the nozzle except for a thin channel, causing the radius *R* to remain almost constant. For $\phi_{m}=0.60$ (dark symbols), *Z* increases linearly with *R* at all volume rates. Buoyancy effects are negligible and the cavity remains attached to the nozzle. For large cavities R>25mm, denoted by open symbols, *Z* increases faster than linear as the walls of the container limit the lateral growth of the cavity. D) Rate of change of *Z* with *R versus* the ratio of the buoyancy velocity and the growth velocity, $V_{b}/V_{g}$. Only data points with R<25mm are shown. When the growth velocity dominates ( $V_{b}/V_{g}<1$), the contribution of buoyancy to the vertical motion is negligible and dZ/dR=0.7 (dash-dot line). When the buoyancy velocity dominates ( $V_{b}/V_{g}>1$), the velocity is set by the balance between buoyancy and viscous forces and $\dot{Z}=V_{b}$ which yields $dZ/dR=0.7V_{b}/V_{g}$ (dashed line).

While the volume rate is unaffected by the cornstarch mass fraction, other aspects of the cavity growth depend sensitively on $\phi_{m}$, starting with the vertical motion of the cavity. For $\phi_{m}=0.57$, the bubble-like cavity detaches from the nozzle and rises upward, as shown in Fig. 2A. This behavior is similar to that observed for a bubble rising in a Newtonian fluid at Reynolds number Re<2 (see Supplementary Text and Fig. S2) (36). By contrast, for $\phi_{m}=0.60$ the air cavity remains connected to the nozzle and the bottom of the cavity does not rise, as shown in Fig. 2B. Since the cavity does not rise, it continues to grow until it breaches the top of the cornstarch suspension, at which point air escapes to the atmosphere.

We quantify the differences between the rising cavities at $\phi_{m}=0.57$ and the growing cavities at $\phi_{m}=0.60$ by measuring the vertical position $Z=\frac{1}{\Omega }\int zd\Omega$ of the center of the cavity, as shown in Fig. 2C. As air is continuously injected, *Z* increases both as a result of the rise and growth of the cavity. For $\phi_{m}=0.57$, *Z* increases faster than the equivalent radius $R=(\frac{3}{4\pi }\Omega {)}^{1/3}$ of the cavity, due to buoyancy forces that act on the negatively buoyant air. The cavity eventually detaches from the nozzle and stops growing, as indicated by gray symbols in Fig. 2C. The buoyancy force acting on the cavity is $F_{b}=\frac{4\pi }{3}R^{3}\Delta \rho g$, where Δρ is the density difference between air and the cornstarch suspension and *g* is the gravitational acceleration (35). The motion of the air cavity is resisted by the viscous drag force $F_{v}=6\pi \eta R\dot{Z}$, where $\dot{Z}$ is the vertical velocity of the cavity and *η* is an effective viscosity of the suspension (36). Balancing the two forces, $F_{b}=F_{v}$, and solving for $\dot{Z}$ yields the cavity velocity in the buoyancy-dominated regime $V_{b}$


(1) $$V_{b}=\frac{2\Delta \rho gR^{2}}{9\eta }.$$

Comparing the measured vertical velocities $\dot{Z}$ with $V_{b}$ for $\phi_{m}=0.57$ yields an estimate for the viscosity η=23Pas, which is an order of magnitude higher than the minimum viscosity measured in the rheometer, as shown in the Methods section. We indeed expect the effective viscosity resisting cavity motion to be larger than the minimum viscosity, as the shear-rate imposed by the cavity motion decreases away from the cavity boundary and the suspension is shear-thinning for low shear rates. Cavities with a higher $P_{g}$, and thus a higher volume rate $\dot{\Omega }$, rise less for the same value of *R*. This can be understood by considering the rate of change of *Z* with *R* in the buoyancy-dominated regime, $dZ/dR=2\Delta \rho gR^{2}/(9\eta \dot{R})$, which decreases with $\dot{R}$ and thus with $\dot{\Omega }$: at a larger volume rate, the buoyancy force acts on the cavity for less time before its volume increases. For $\phi_{m}=0.60$, the vertical position *Z* increases slower than the equivalent radius *R* of the cavity and the *Z* versus *R* curves are independent of the volume rate set by $P_{g}$. The stagnation of the bottom of the cavity indicates that buoyancy forces are not at play: for this suspension with high mass fraction, the large effective viscosity leads to $F_{v}\gg F_{b}$. In the absence of buoyancy and for a cavity growing while maintaining a similar shape, we indeed expect Z∼R independently of the volume rate. The cavity vertical velocity in the growth-dominated regime $V_{g}$ is


(2) $$V_{g}=a\dot{R},$$

where a≈0.7 is a geometric parameter that varies slightly depending on the shape of the cavity. We observe that for R>25mm (indicated by open symbols in Fig. 2), the rate of change of *Z* with *R* increases, even though the cavity remains attached to the nozzle. This increase is due to the walls of the container limiting lateral growth as the air cavity gets close to the walls, leading to enhanced vertical growth.

At an intermediate mass fraction of $\phi_{m}=0.58$, the air cavity dynamically transitions between the growth-dominated and the buoyancy-dominated regimes, as shown in Fig. S3. As the volume rate is constant, $R\propto t^{1/3}$ and the vertical velocity due to growth $V_{g}=a\dot{R}$ decreases with *R* while the vertical velocity due to buoyancy increases with *R*. The quasistatic growth-dominated regime and the buoyancy-dominated regime correspond to two asymptotes in the dZ/dR*versus*$V_{b}/V_{g}$ curves shown in Fig. 2D, where $V_{b}/V_{g}=\frac{2\Delta \rho gR^{2}}{9\eta \dot{R}}$ is the ratio of the velocities predicted in the two regimes for the measured *R* and $\dot{R}$. The transition occurs for $V_{b}/V_{g}=1$. Despite the complex rheology of the suspension, a single value of *η* captures the vertical motion at $\phi_{m}=0.57$ and 0.58, which indicates that the suspension is in the lubricated regime where the viscosity shows only a weak shear-thinning behavior.

### Transition from smooth bubbles to fractures

**Fig. 3.**
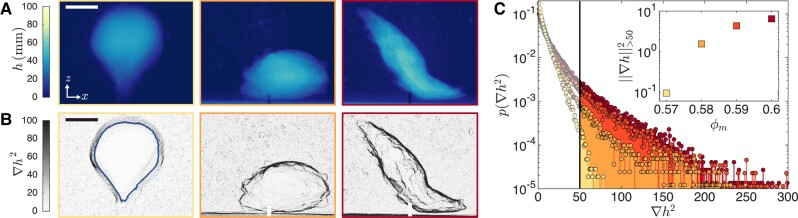
Fracture characteristics at cavity interface. A) Air thickness at a cavity volume Ω=50mL for cornstarch mass fractions, from left to right, $\phi_{m}=0.57,0.58,0.60$ and $P_{g}=20\mathrm{kPa}$. B) Intensity of the air thickness gradient $\nabla h^{2}=(\partial h/\partial x{)}^{2}+(\partial h/\partial z{)}^{2}$. Darker lines correspond to local fractures at the cornstarch at the cavity interface. For the analysis, we shrink the cavity outline by 5% in radius to define a region of interest that does not include the effect of the cavity edges on the thickness gradient (denoted by a blue line in the $\phi_{m}=0.57$ image). The scale bars represent 2 cm. C) Histogram of the air thickness gradient values inside the region of interest. The proportion of large values of $\nabla h^{2}$ corresponding to sharp features increases with mass fraction, which indicates a transition from smooth to fractured cavities. Inset: Metric for local fracture characterization $\|\nabla h{\|}_{>50}^{2}=\int_{50}^{\infty }p(\nabla h^{2})d\nabla h^{2}$ versus mass fraction $\phi_{m}$. Performing the integration over the entire range of $\nabla h^{2}$ would yield the square of the norm of ∇h. We integrate from 50 to accentuate the effect of sharp features on the metric. The lower bound of the integral does not affect the observed trend, as shown in Fig. S4.

Contrary to the smooth bubbles observed in Newtonian or viscoplastic liquids (36, 39, 56), air injection into dense cornstarch suspensions can lead to fractures. In the early stage of air injection, a fracture reminiscent of the thin cracks that form during liquid injection into elastic solids occurs (42, 43), as seen in the Supplementary Movie S1. Instead of propagating further as a thin crack, however, the fracture widens into a three-dimensional cavity. While cavities at lower mass fractions tend to be round and cavities at $\phi_{m}=0.60$ tend to be elongated, the precise shape of the cavity depends on the initial orientation of the fracture and can vary for experiments performed under the same conditions. Very consistently though, cavities at $\phi_{m}=0.57$ are smooth bubbles, while cavities at $\phi_{m}=0.60$ exhibit fracture characteristics and large local variations in thickness, as shown in Fig. 3A for cavities at Ω=50mL. To visualize this transition from smooth to fractured cornstarch/air interfaces, we measure the gradient of the air thickness and show the thickness gradient intensity $\nabla h^{2}=(\partial h/\partial x{)}^{2}+(\partial h/\partial z{)}^{2}$ for experiments at $P_{g}=20\mathrm{kPa}$ in Fig. 3B (Movie S2). The thickness gradient intensity images reveal sharp elongated features for $\phi_{m}\geq 0.58$. We attribute these features to local fracturing of the cornstarch suspension.

To quantify the characteristics of the local fractures, we measure the histogram of the thickness gradient intensity $\nabla h^{2}$ inside the cavity. Because the edges of the cavity where h→0 correspond to regions of high gradient intensity even for smooth bubbles, we evaluate the thickness gradient intensity in a smaller region obtained by shrinking the cavity outline by 5% in radius, as shown for the bubble formed in the $\phi_{m}=0.57$ suspension in Fig. 3B. With increasing mass fraction, the proportion of high values of $\nabla h^{2}$ corresponding to sharp features increases, as seen in the histograms in Fig. 3C. We define a metric for local fracture characterization, $\|\nabla h{\|}_{>50}^{2}=\int_{50}^{\infty }p(\nabla h^{2})d\nabla h^{2}$, which quantifies the amount of sharp features with $\nabla h^{2}>50$ (our conclusions are robust to the choice of this cut off value, see the Supplementary Text and Fig. S4). $\|\nabla h{\|}_{>50}^{2}$ increases with $\phi_{m}$, as displayed in the inset of Fig. 3C, and also increases with $P_{g}$ for a fixed $\phi_{m}$, as shown in the Supplementary Text and Fig. S5.

Fracturing occurs in solid materials. The fractures seen in Fig. 3 thus show that the suspension is in a SJ state during the growth of the cavity. Indeed, all the suspensions used in this study can exhibit DST and SJ. The onset of DST measured in a rheometer occurs at a critical shear rate ${\dot{\gamma }}_{c}$, while SJ occurs at higher stresses and sufficient accumulated strain (8, 16, 26, 29). The gas tank pressure $P_{g}$ sets the maximum stress applied to the suspension, and fractures occur only for pressures above 5kPa. At a lower pressure of $P_{g}=2\mathrm{kPa}$, no air enters the suspension at $\phi_{m}=0.60$ and a smooth bubble-like cavity forms at lower mass fractions. The growth of an air cavity with equivalent radius *R* deforms the suspension, inducing a shear rate that is largest at the cavity interface. As the cavity expands, a volume of suspension in contact with the cavity is stretched thinner in the *r*-direction of cavity expansion and wider in the *θ*- and *ϕ*-directions along the interface of the cavity. For a growing spherical cavity, the shear rates at the interface are $S_{rr}=-2\dot{R}/R$ and $S_{\theta \theta }=S_{\phi \phi }=\dot{R}/R$ (36, 48, 57). As shown in the Supplementary Text, the expression for $S_{rr}$ remains valid for a cavity of any shape with $R=(\frac{3}{4\pi }\Omega {)}^{1/3}$ as long as the cavity maintains its shape as it expands, which is the case for most of the cavity growth. At high mass fractions, this local shear rate is larger than ${\dot{\gamma }}_{c}$. The suspension around the cavity enters a SJ state and sliding cracks form at the interface of the cavity to accommodate the shear rate, creating variations in thickness reflected in the high value of $\|\nabla h{\|}_{>50}^{2}$. At first sight it might then surprise that the air bubbles at lower mass fraction do not exhibit sharp features at Ω=50mL despite the suspension being able to exhibit DST and SJ. However, we need to consider that the shear rate $S_{rr}=-2\dot{R}/R$ decreases during the cavity growth and that ${\dot{\gamma }}_{c}$ increases with decreasing mass fraction. This allows for early fracture followed by relaxation.

### Cavity interface relaxation during growth

**Fig. 4.**
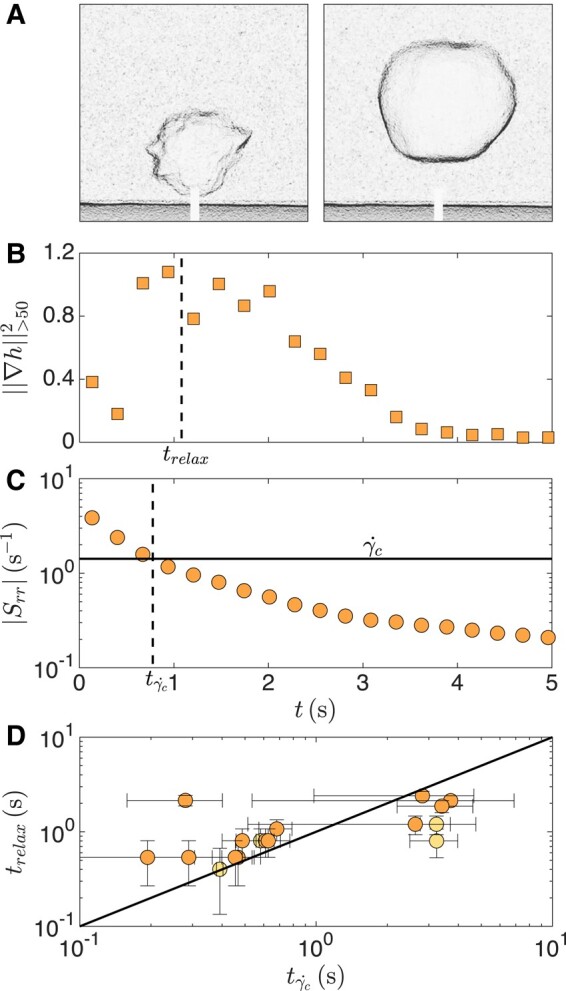
Relaxation of the cornstarch suspension around the air cavity. A) Gradient intensity images at t=1.1s and 3.8 s show the relaxation for $\phi_{m}=0.58$ and $P_{g}=60\mathrm{kPa}$. The scale bar represents 2 cm. B) The metric for local fracture characterization $\|\nabla h{\|}_{>50}^{2}$ decreases beyond the time $t_{\mathrm{relax}}$, indicating that the cornstarch surrounding the cavity relaxes. C) The shear rate $|S_{rr}|=2\dot{R}/R$ induced by the cavity growth decreases as the cavity grows and falls below the critical shear rate denoting the onset of DST ${\dot{\gamma }}_{c}=1.4s^{-1}$ (horizontal line) at $t_{\dot{\gamma_{c}}}$ indicated by the vertical dashed line. D) The time denoting the onset of relaxation $t_{\mathrm{relax}}$ is consistent with the time $t_{\dot{\gamma_{c}}}$ at which $S_{rr}={\dot{\gamma }}_{c}$. The fairly large uncertainties in $t_{\dot{\gamma_{c}}}$ stem from the uncertainty in the value of ${\dot{\gamma }}_{c}$ measured in rheology experiments (Fig. 5).

Indeed, the cavities forming in suspensions with $\phi_{m}=0.57$ and 0.58 fracture at early times when the shear rate induced by the cavity growth is largest, but subsequently relax to smooth bubbles, as seen in the thickness gradient intensity images shown in Fig. 4A. This relaxation of the interface is well captured by the time evolution of $\|\nabla h{\|}_{>50}^{2}$, which shows that the suspension surrounding the cavity begins to relax at a time $t_{\mathrm{relax}}$, as seen in Fig. 4B. We calculate the shear rate at the cavity interface during cavity growth, $S_{rr}=-2\dot{R}/R$ (36, 48, 57). $S_{rr}$ decreases as the cavity grows, as seen in Fig. 4C, and reaches a value below the critical shear rate denoting the onset of DST ${\dot{\gamma }}_{c}$ at a critical time denoted $t_{\dot{\gamma_{c}}}$. Remarkably, $t_{\dot{\gamma_{c}}}$ almost coincides with the time $t_{\mathrm{relax}}$ at which the cavity shape starts to relax. Such correlation between the two time scales is generally observed for experiments at $\phi_{m}=0.57$ and 0.58, as shown in Fig. 4D. The relaxation of the cavity interface when $S_{rr}<{\dot{\gamma }}_{c}$ shows that the stress applied to the suspension has fallen below the critical stress for shear jamming and that the sharp features are a structural signature of the SJ state.

## Discussion

We demonstrate the rich dynamics of cavity growth occurring as air is injected into bulk cornstarch suspensions. Smooth bubbles that form in suspensions with cornstarch mass fraction $\phi_{m}\leq 0.57$ rise up and detach from the nozzle similar to the behavior observed in Newtonian baths. The relative importance of buoyancy decreases with increasing $\phi_{m}$, leading to a quasistatic growth regime in which air cavities expand without rising. The suspensions used in this study exhibit DST in the rheometer and fracture when violently handled, indicating that they can reach a SJ state. Remarkably, we discover a structural signature of the SJ state by considering the shape of the cavity interface. The cornstarch/air interface exhibits local fracture characteristics that we quantify by defining the metric for local fracture characterization $\|\nabla h{\|}_{>50}^{2}$ which increases with $\phi_{m}$. We rationalize this observation by considering the expansion of an air cavity with equivalent radius *R*, which deforms the suspension directly surrounding the air cavity with a shear rate $S_{rr}=-2\dot{R}/R$. During the early times of cavity growth, this shear rate is higher than the critical shear rate ${\dot{\gamma }}_{c}$ denoting the onset of discontinuous shear thickening of the suspension, and the suspension fractures. The fracturing of the suspension is related to the vertical motion of the cavity. Sharp features in the cornstarch suspension indicate a SJ state with a very high effective viscosity. Consequently, a large drag force opposes buoyancy and the vertical motion of the cavity is dominated by growth. Conversely, the rise of the air cavity under buoyancy only occurs in a relaxed suspension. We hypothesize that with increasing cornstarch mass fraction $\phi_{m}$, the increase in the discrepancy between the imposed shear rate $\left|S_{rr}\right|$ and ${\dot{\gamma }}_{c}$ triggers more sliding cracks in the suspension to accommodate cavity expansion, resulting in the observed increase in $\|\nabla h{\|}_{>50}^{2}$. Confirming this hypothesis, increasing $\left|S_{rr}\right|$ while keeping ${\dot{\gamma }}_{c}$ the same by varying the gas tank pressure $P_{g}$ also results in an increase in $\|\nabla h{\|}_{>50}^{2}$, as shown in Fig. S5.

It is interesting to compare the fracture formation and relaxation in dense cornstarch suspensions to those observed in wormlike micellar solutions that exhibit brittle viscoelastic behavior. Wormlike micellar solutions have a storage modulus that is constant with frequency and dominates over the loss modulus for shear rates larger than the inverse of the relaxation time of the micelles network, 1/τ (48, 58). Air injected into this material at constant volume rate induces a shear rate $|S_{rr}|=2\dot{R}/R>1/\tau$ at early times. The response of the material is thus initially elastic and the stress increases with the accumulated strain. If the critical stress for fracture is reached before the shear rate decreases below 1/τ, the material fractures. Consequently, the bubble initially grows smoothly until the critical stress for fracture is reached, and fracture is only observed after a certain delay time. By comparison, dense cornstarch suspensions fracture almost immediately under air injection. This is because after only a small initial deformation, the viscosity of the suspension diverges if the applied shear rate is larger than ${\dot{\gamma }}_{c}$ (29). High strain-rate experiments where a rod impacts a thin layer of cornstarch suspension showed that, when the impact is sufficiently violent to fracture the suspension, the fracture occurs for a strain ε≈0.12 (9). In our experiments, since the shear rates are initially high, the strain ε≈dR/R increases above 0.12 within the first two X-ray images, which corresponds to the observed initiation of fracture. Since the applied pressure and the shear rate are both highest at the beginning of air injection, the suspension shear jams and fractures at an early stage. Another interesting comparison is that between a dense suspension and a solid material. Fluid injection into a solid material creates a thin fracture perpendicular to the injection nozzle that then expands in the radial direction, as elastic stresses prevent the fracture from widening (41, 44). The fractures we observe in dense cornstarch suspensions are initially thin as the suspension behaves like a solid, but then widen as the suspension away from the air/suspension interface is able to flow.

Lastly, our results offer insights into the dynamic transition of a dense suspension in and out of a SJ state. Dense suspensions enter a SJ state when subjected to a large enough stress (19, 26). We find that a minimum pressure of 5 kPa is necessary to form fractures, a value that agrees well with extensional rheology experiments where a $\phi_{m}=0.55$ cornstarch suspension fractured at a tensile stress of 10 kPa (11). Conversely, the relaxation out of a SJ state occurs when two conditions are met: (i) the shear rate is lower than the critical shear rate denoting the onset of DST and (ii) the stress is lower than the critical stress for shear jamming (28, 31, 33). For the lower range of $\phi_{m}$ investigated, the cavities relax once the shear rate at the cavity interface becomes smaller than ${\dot{\gamma }}_{c}$. For the highest range of mass fractions investigated, we did not observe relaxation even after $S_{rr}<{\dot{\gamma }}_{c}$. This indicates that the stress applied to the suspension remains larger than the critical stress for shear jamming. As this stress is related to the air pressure inside the cavity, it will be interesting to measure the pressure at the nozzle inlet as a proxy for the pressure inside the cavity in follow-up work. Simultaneous measurements of the stress via the air pressure and the shear rate via X-ray imaging would further allow to estimate an effective suspension viscosity during the growth of the cavity (48).

## Materials and methods

### Sample preparation

Cornstarch suspensions are prepared by mixing cornstarch particles (Sigma-Aldrich) and deionized water by hand until the suspension is homogeneous. A lid on the container used in the X-ray radiography experiments limits evaporation and the one kilogram cornstarch suspensions are reused in up to three experiments before preparing a new suspension. Between each experiment, the cornstarch suspension is taken out of the container and poured into a different container to remove any memory of the previous experiment, ensure proper mixing and avoid excessive settling of the particles. The container is agitated to promote the rise of any small air bubbles present in the suspension before being placed in the X-ray scanner. The time between the pouring of the suspension into the container and the beginning of air injection is kept under five minutes.

### Rheology

**Fig. 5.**
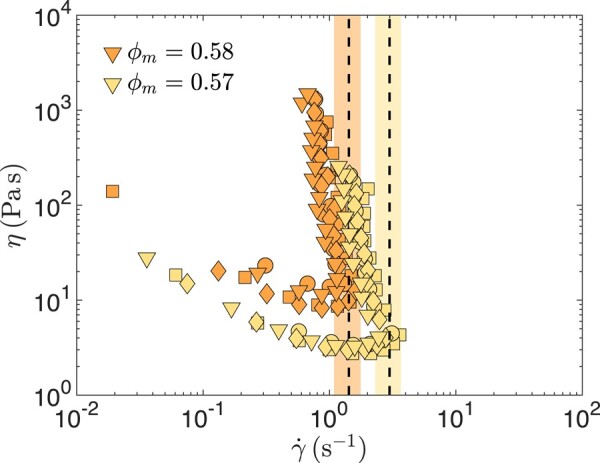
Steady-state apparent viscosity *η* of dense cornstarch suspensions with mass fractions $\phi_{m}$ as a function of shear rate $\dot{\gamma }$. The four symbols correspond to different stress sweeps in the rheology experiment. The critical shear rate ${\dot{\gamma }}_{c}$ is determined as the maximum shear rate. The mean value and 95% uncertainty of ${\dot{\gamma }}_{c}$ are indicated with a dashed line and a shaded region.

The stress-dependent viscosities of the cornstarch suspensions are measured using stress-controlled rheometers (DHR-3 and AR-G2, TA Instruments) with a 40 mm diameter parallel plate geometry and a gap spacing of 1,250μm. Sandpaper (grit size: P120, 102μm in diameter) is used on both plates to prevent wall slip. The top plate of the geometry is lowered slowly and the geometry is rotated back and forth while being lowered to ensure that the normal compression force remains below 2 N. The sample is then presheared at a shear rate of $0.01s^{-1}$ for 30 s and equilibrated at zero stress for 30 s. Stress sweeps are conducted with the shear stress first decreasing from 1,000 Pa to 1 Pa, then increasing back to 1,000 Pa, and repeating the process for a total of two decreasing and two increasing sweeps, as shown in Fig. 5.

### X-ray radiography

Air injection is visualized using an X-ray CT scanner (Nikon XT H 225) with a tungsten target excited at 200 kV and a current of 150μA. To obtain the air thickness from the transmitted X-ray intensity data, we measure the X-ray intensity transmitted through containers of varying thicknesses filled with cornstarch suspensions. The transmitted intensity is independent of the mass fraction of cornstarch in the range used in this study, and can be fit to the Beer–Lambert equation (54):


(3) $$I=I_{0}\cdot \exp\;(-l/\lambda )+I_{c},$$

where $I_{0}$ is the intensity transmitted through the empty acrylic container, $I_{c}\approx 0.05I_{0}$ is a small constant term, *l* is the thickness of the cornstarch suspension and λ=35mm is the inverse of the attenuation coefficient. Knowing the dimensions of the acrylic container, 100mm×100mm×150mm, the air thickness is obtained as h=100mm−l.

## Supplementary Material

pgad451_Supplementary_Data

## Data Availability

The data underlying this article are available in the University of Southampton Institutional Research Repository at https://eprints.soton.ac.uk/ and can be accessed at https://doi.org/10.5258/SOTON/D2920. Due to the large size of the X-ray files, the raw data will be made available upon request to Paul Lilin plilin@mit.edu.

## Previous Presentation

These results were previously presented at the APS DFD Meeting in November 2023.

## References

[1] Hoffman RL . 1972. Discontinuous and dilatant viscosity behavior in concentrated suspensions. I. Observation of a flow instability. Trans Soc Rheol. 16(1):155–173.

[2] Barnes HA . 1989. Shear-thickening (“dilatancy”) in suspensions of nonaggregating solid particles dispersed in newtonian liquids. J Rheol. 33(2):329–366.

[3] Boersma WH , Laven J, Stein HN. 1990. Shear thickening (dilatancy) in concentrated dispersions. AICHE J. 36(3):321–332.

[4] Denn MM , Morris JF. 2014. Rheology of non-Brownian suspensions. Annu Rev Chem Biomol Eng. 5(1):203–228. 24655134 10.1146/annurev-chembioeng-060713-040221

[5] Brown E , Jaeger HM. 2009. Dynamic jamming point for shear thickening suspensions. Phys Rev Lett. 103(8):086001. 19792740 10.1103/PhysRevLett.103.086001

[6] Seto R , Mari R, Morris JF, Denn MM. 2013. Discontinuous shear thickening of frictional hard-sphere suspensions. Phys Rev Lett. 111(21):218301. 24313532 10.1103/PhysRevLett.111.218301

[7] Mari R , Seto R, Morris JF, Denn MM. 2015. Discontinuous shear thickening in Brownian suspensions by dynamic simulation. Proc Natl Acad Sci USA. 112(50):15326–15330. 26621744 10.1073/pnas.1515477112 PMC4687578

[8] Morris JF . 2020. Shear thickening of concentrated suspensions: recent developments and relation to other phenomena. Annu Rev Fluid Mech. 52(1):121–144.

[9] Roché M , Myftiu E, Johnston MC, Kim P, Stone HA. 2013. Dynamic fracture of nonglassy suspensions. Phys Rev Lett. 110(14):148304. 25167046 10.1103/PhysRevLett.110.148304

[10] Allen B , Sokol B, Mukhopadhyay S, Maharjan R, Brown E. 2018. System-spanning dynamically jammed region in response to impact of cornstarch and water suspensions. Phys Rev E. 97(5):052603. 29906931 10.1103/PhysRevE.97.052603

[11] Bischoff White EE , Chellamuthu M, Rothstein JP. 2010. Extensional rheology of a shear-thickening cornstarch and water suspension. Rheol Acta. 49(2):119–129.

[12] Wang Z-Y , Zhao H, Li W-F, Xu J-L, Liu H-F. 2022. Breakup of shear-thickening suspension. Phys Rev Fluids. 7(7):073601.

[13] Ozturk D , Morgan ML, Sandnes B. 2020. Flow-to-fracture transition and pattern formation in a discontinuous shear thickening fluid. Commun Phys. 3(1):1–9.

[14] Niu R , Ramaswamy M, Ness C, Shetty A, Cohen I. 2020. Tunable solidification of cornstarch under impact: how to make someone walking on cornstarch sink. Sci Adv. 6(19):eaay6661. 32494699 10.1126/sciadv.aay6661 PMC7209985

[15] Fall A , Huang N, Bertrand F, Ovarlez G, Bonn D. 2008. Shear thickening of cornstarch suspensions as a reentrant jamming transition. Phys Rev Lett. 100(1):018301. 18232829 10.1103/PhysRevLett.100.018301

[16] Fall A , Bertrand F, Ovarlez G, Bonn D. 2012. Shear thickening of cornstarch suspensions. J Rheol. 56(3):575–591. 10.1103/PhysRevLett.100.018301 18232829

[17] Crawford NC , *et al*. 2013. Shear thickening of corn starch suspensions: does concentration matter? J Colloid Interface Sci. 396:83–89. 23484772 10.1016/j.jcis.2013.01.024

[18] Hermes M , *et al*. 2016. Unsteady flow and particle migration in dense, non-Brownian suspensions. J Rheol. 60(5):905–916.

[19] Wyart M , Cates ME. 2014. Discontinuous shear thickening without inertia in dense non-Brownian suspensions. Phys Rev Lett. 112(9):098302. 24655284 10.1103/PhysRevLett.112.098302

[20] Mari R , Seto R, Morris JF, Denn MM. 2014. Shear thickening, frictionless and frictional rheologies in non-Brownian suspensions. J Rheol. 58(6):1693–1724.

[21] Clavaud C , Bérut A, Metzger B, Forterre Y. 2017. Revealing the frictional transition in shear-thickening suspensions. Proc Natl Acad Sci USA. 114(20):5147–5152. 28465437 10.1073/pnas.1703926114 PMC5441771

[22] Ovarlez G , *et al*. 2020. Density waves in shear-thickening suspensions. Sci Adv. 6(16):eaay5589. 32494596 10.1126/sciadv.aay5589 PMC7164946

[23] Royer JR , Blair DL, Hudson SD. 2016. Rheological signature of frictional interactions in shear thickening suspensions. Phys Rev Lett. 116(18):188301. 27203345 10.1103/PhysRevLett.116.188301

[24] Bi D , Zhang J, Chakraborty B, Behringer RP. 2011. Jamming by shear. Nature. 480(7377):355–358. 22170683 10.1038/nature10667

[25] Brown E , Jaeger HM. 2014. Shear thickening in concentrated suspensions: phenomenology, mechanisms and relations to jamming. Rep Prog Phys. 77(4):046602. 24695058 10.1088/0034-4885/77/4/046602

[26] Peters IR , Majumdar S, Jaeger HM. 2016. Direct observation of dynamic shear jamming in dense suspensions. Nature. 532(7598):214–217. 27042934 10.1038/nature17167

[27] James NM , Xue H, Goyal M, Jaeger HM. 2019. Controlling shear jamming in dense suspensions via the particle aspect ratio. Soft Matter. 15(18):3649–3654. 30994148 10.1039/c9sm00335e

[28] Baumgarten AS , Kamrin K. 2020. Modeling stress relaxation in dense, fine-particle suspensions. J Rheol. 64(2):367–377.

[29] Han E , Wyart M, Peters IR, Jaeger HM. 2018. Shear fronts in shear-thickening suspensions. Phys Rev Fluids. 3(7):073301.

[30] Rathee V , Miller J, Blair DL, Urbach JS. 2022. Structure of propagating high-stress fronts in a shear-thickening suspension. Proc Natl Acad Sci USA. 119(32):e2203795119. 10.1073/pnas.2203795119 PMC9371692 35914166

[31] Maharjan R , Brown E. 2017. Giant deviation of a relaxation time from generalized newtonian theory in discontinuous shear thickening suspensions. Phys Rev Fluids. 2(12):123301.

[32] Barik S , Majumdar S. 2022. Origin of two distinct stress relaxation regimes in shear jammed dense suspensions. Phys Rev Lett. 128(25):258002. 35802438 10.1103/PhysRevLett.128.258002

[33] Cho JH , Griese AH, Peters IR, Bischofberger I. 2022. Lasting effects of discontinuous shear thickening in cornstarch suspensions upon flow cessation. Phys Rev Fluids. 7(6):063302.

[34] Dekée D , Carreau PJ, Mordarski J. 1986. Bubble velocity and coalescence in viscoelastic liquids. Chem Eng Sci. 41(9):2273–2283.

[35] Davidson JF , Schüler BOG. 1997. Bubble formation at an orifice in a viscous liquid. Chem Eng Res Des. 75:S105–S115.

[36] Kulkarni AA , Joshi JB. 2005. Bubble formation and bubble rise velocity in gas-liquid systems: a review. Ind Eng Chem Res. 44(16):5873–5931.

[37] Sun B , *et al*. 2015. Experimental study on the drag coefficient of single bubbles rising in static non-newtonian fluids in wellbore. J Nat Gas Sci Eng. 26:867–872.

[38] Samson G , Phelipot-Mardelé A, Lanos C, Pierre A. 2017. Quasi-static bubble in a yield stress fluid: elasto-plastic model. Rheol Acta. 56:1–13.

[39] Lopez WF , Naccache MF, de Souza Mendes PR. 2018. Rising bubbles in yield stress materials. J Rheol. 62(1):209–219.

[40] Zhao K , Tedford EW, Zare M, Frigaard IA, Lawrence GA. 2021. Bubbles rising through a layer of Carbopol capped with water. J Non-Newton Fluid Mech. 300:104700.

[41] Hubbert MK , Willis DG. 1957. Mechanics of hydraulic fracturing. Petrol Trans. 210(01):153–168.

[42] Takada A . 1990. Experimental study on propagation of liquid-filled crack in gelatin: shape and velocity in hydrostatic stress condition. J Geophys Res Solid. 95(B6):8471–8481.

[43] Lai C-Y , Zheng Z, Dressaire E, Wexler JS, Stone HA. 2015. Experimental study on penny-shaped fluid-driven cracks in an elastic matrix. Proc Math Phys Eng. 471(2182):20150255.

[44] Lai C-Y , *et al*. 2016. Elastic relaxation of fluid-driven cracks and the resulting backflow. Phys Rev Lett. 117(26):268001. 28059547 10.1103/PhysRevLett.117.268001

[45] Pilz C , Brenn G. 2007. On the critical bubble volume at the rise velocity jump discontinuity in viscoelastic liquids. J Non-Newton Fluid Mech. 145(2):124–138.

[46] Mora S , Manna M. 2012. From viscous fingering to elastic instabilities. J Non-Newtonian Fluid Mech. 173–174:30–39.

[47] Foyart G , Ramos L, Mora S, Ligoure C. 2013. The fingering to fracturing transition in a transient gel. Soft Matter. 9(32):7775–7779.

[48] Sánchez C , Ichihara M, Kuwano O. 2023. Flow-to-fracture transition of linear Maxwell-type versus yield strength fluids by air injection—Implications for magma fracturing. Geophys Res Lett. 50(1):e2022GL100918.

[49] Du L , Folliard KJ. 2005. Mechanisms of air entrainment in concrete. Cement Concrete Res. 35(8):1463–1471.

[50] Petit P , Javierre I, Jézéquel P-H, Biance A-L. 2014. Generation and stability of bubbles in a cement based slurry. Cement Concrete Res. 60:37–44.

[51] Stewart RB , Schouten FC. 1988. Gas invasion and migration in cemented annuli: causes and cures. SPE Drilling Eng. 3(01):77–82.

[52] Tran A , Rudolph ML, Manga M. 2015. Bubble mobility in mud and magmatic volcanoes. J Volcanol Geoth Res. 294:11–24.

[53] Parmigiani A , Faroughi S, Huber C, Bachmann O, Su Y. 2016. Bubble accumulation and its role in the evolution of magma reservoirs in the upper crust. Nature. 532(7600):492–495. 27074507 10.1038/nature17401

[54] Als-Nielsen J , McMorrow D. 2011. Elements of modern X-ray physics. Chichester: John Wiley & Sons.

[55] Ramakrishnan S , Kumar R, Kuloor NR. 1969. Studies in bubble formation—I bubble formation under constant flow conditions. Chem Eng Sci. 24(4):731–747.

[56] Margaritis A , te Bokkel DW, Karamanev DG. 1999. Bubble rise velocities and drag coefficients in non-newtonian polysaccharide solutions. Biotechnol Bioeng. 64(3):257–266. 10397862 10.1002/(sici)1097-0290(19990805)64:3<257::aid-bit1>3.0.co;2-f

[57] Barlow EJ , Langlois WE. 1962. Diffusion of gas from a liquid into an expanding bubble. IBM J Res Dev. 6(3):329–337.

[58] Arora S , Shabbir A, Hassager O, Ligoure C, Ramos L. 2017. Brittle fracture of polymer transient networks. J Rheol. 61(6):1267–1275.

