# Supplementary material for: Fracture and relaxation in dense cornstarch suspensions
Source: PNAS Nexus. 2023 Dec 22;3(1):pgad451. doi: 10.1093/pnasnexus/pgad451 (PMC10785035; doi:10.1093/pnasnexus/pgad451)
Supplement: pgad451_Supplementary_Data [file pgad451_supplementary_data.zip › PNASNEXUS-PNASNEXUS-2023-01235-T-s05.pdf]

PNAS nexus

Supporting Information for

Fracture and relaxation in dense cornstarch suspensions

Paul Lilin, Jean E. Elkhoury, Ivo R. Peters, Irmgard Bischofberger

Paul Lilin, Irmgard Bischofberger.
E-mail: plilin@mit.edu, irmgard@mit.edu

This PDF file includes:

Supporting text
Figs. S1 to S5
Legends for Movies S1 to S2
SI References

Other supporting materials for this manuscript include the following:

Movies S1 to S2

Supporting Information Text

1. Growth dynamics of air bubbles in viscous Newtonian liquids

We investigate the pressure losses in the system and the growth of bubbles by considering air injection into Newtonian liquids of varying viscosities. Air injection into a viscous silicone oil (viscosity $\eta = 70\,\mathrm{Pa\,s}$) occurs at constant volume rates $\dot{\Omega}$ that increase linearly with the pressure difference between the gas tank and the liquid at the height of the nozzle $P_g - P_h$, as shown in fig. S1A and B. The volume rate is independent of the viscosity η of the Newtonian fluid, as seen when comparing the bubble growth in the silicone oil with that in glycerol ($\eta = 1.4\,\mathrm{Pa\,s}$). This indicates that the pressure is lost in the tubing between the gas tank and the nozzle: $P_g - P_b = R_h\dot{\Omega}$, with P_b the pressure in the bubble and R_h the hydraulic resistance of the tubing.

For air injection into a Newtonian liquid, the pressure loss at the bubble interface due to viscous dissipation in the liquid is $P_b - P_h = \frac{2}{3}\eta\frac{\dot{\Omega}_i}{\Omega}$ with $\dot{\Omega}_i$ the instantaneous volume rate (1). Since this pressure loss decreases with the bubble volume Ω, it has an effect for small bubble volumes only. In fig. S1C, we report the instantaneous volume rate divided by the gas tank gauge pressure $P_g - P_h$ versus the bubble volume Ω for air injection into silicone oil. For bubble volumes below 0.07 mL, the viscous pressure loss dominates: $P_b \approx P_g$ and $\dot{\Omega}_i/(P_g - P_h) = 3\Omega/(2\eta)$. The majority of the bubble growth, however, occurs at a constant volume rate where $P_b \approx P_h$ and $\dot{\Omega}_i/(P_g - P_h) = 1/R_h$. This is confirmed by measuring P_g and the pressure at the inlet of the nozzle $P_{nozzle} \approx P_b$, as shown in fig. S1D. The bubble pressure is initially equal to the gas tank pressure, but quickly drops to lower values as the bubble grows.

2. Vertical motion of air bubbles in viscous Newtonian liquids

The process outlined in the main manuscript to capture the vertical motion of air bubbles injected into cornstarch suspensions is here applied to air bubbles injected into Newtonian liquids. Air bubbles injected into glycerol (viscosity $\eta = 1.4\,\mathrm{Pa\,s}$) at $P_g = 20\,\mathrm{kPa}$ detach from the nozzle within 0.2 s, as seen in fig. S2A. In the more viscous silicone oil ($\eta = 70\,\mathrm{Pa\,s}$), the vertical motion of the bubble is slowed down by viscous drag and the bubble is still attached and growing at $t = 1.6\,\mathrm{s}$, as seen in fig. S2B.

Bubbles in glycerol rise and detach for smaller equivalent radii R than bubbles in silicone oil, as shown in fig. S2C. In addition, dZ/dR decreases as P_g increases, where Z is the vertical position of the center of the bubble. Balancing the buoyancy force $F_b = 4\pi\Delta\rho g R^3/3$ with the viscous drag $F_v = 6\pi\eta R\dot{Z}$ yields $V_b = 2\Delta\rho g R^2/(9\eta)$, where $\Delta\rho$ is the density difference between air and the liquid and g is the gravitational acceleration. This expression can be rewritten as $dZ/dR = 2\Delta\rho g R^2/(9\eta\dot{R})$. Increasing P_g leads to a faster bubble growth, thus a larger radius growth velocity $\dot{R}$ and a smaller dZ/dR, as observed experimentally.

For small bubble volumes, the viscous drag $F_v \propto R$ is much larger than the buoyancy force $F_b \propto R^3$. The upwards motion of the center of the bubble is not due to buoyancy, but due to the directed growth of the bubble out of the air nozzle. In this quasistatic growth-dominated regime, we predict $V_g = a\dot{R}$ and $dZ/dR = a$ with $a \approx 0.7$ from experimental data. Both the quasistatic growth-dominated regime and the buoyancy-dominated regime are observed, depending on the value of $V_b/V_g = 2\Delta\rho g R^2/(9a\eta\dot{R})$, and all data for different flow rates and fluid viscosities collapse, as shown in fig. S2D.

3. Dependence of the metric for local fracture characterization $||\nabla h^2||_{>threshold}$ on the threshold value

The metric for local fracture characterization $||\nabla h^2||_{>50} = \int_{50}^{\infty} p(\nabla h^2)\mathrm{d}\nabla h^2$ used in the main manuscript uses a threshold value of $\nabla h^2 = 50$. This threshold value, or lower bound of the integral, is employed to accentuate the effect of the high gradient content on the metric by removing low gradient content, as shown in fig. S4A.

To ensure that the metric is robust, we vary the threshold value from 30 to 70. The resulting change in $||\nabla h||^2_{>threshold} = \int_{threshold}^{\infty} p(\nabla h^2)\mathrm{d}\nabla h^2$ is small and the metric always increases monotonically with cornstarch mass fraction ϕ_m, as seen in fig. S4B. Increasing the threshold value decreases $||\nabla h||^2_{>threshold}$, since the integration is performed over a smaller range. The metric is most sensitive to the threshold value for $\phi_m = 0.57$ where the cavity is smooth and contains almost no pixels with $\nabla h^2 > 70$, as seen in fig. S4A.

4. Dependence of the metric for local fracture characterization $||\nabla h^2||_{>50}$ on the gas tank pressure

In the main manuscript, we suggest that for an increase in cornstarch mass fraction ϕ_m, the increase in the discrepancy between the imposed shear rate S_{rr} and the shear rate denoting the onset of discontinuous shear thickening $\dot{\gamma}_c$ caused by decreasing $\dot{\gamma}_c$ triggers more sliding cracks in the suspension to accommodate cavity expansion, resulting in the observed increase in $||\nabla h||^2_{>50}$ in Fig. 3. Another way to increase the discrepancy between S_{rr} and $\dot{\gamma}_c$ is to increase $|S_{rr}|$. We achieve this by varying the gas tank pressure P_g, which sets the volume rate and thus the shear rate as $S_{rr} \propto P_g/\Omega$. We observe an increase in high gradient content and thus in $||\nabla h||^2_{>50}$ with increasing P_g for mass fractions $\phi_m \geq 0.59$, as seen in fig. S5A and B.

5. Shear rate at the cavity boundary

A spherical bubble of radius R growing in an infinite bath of fluid induces a purely radial flow $u_r(r) = \frac{R^2}{r^2}\dot{R}$ that corresponds to the shear rates $S_{rr}(r) = -2\frac{R^2}{r^3}\dot{R}$ in the radial direction, and $S_{\theta\theta}(r) = S_{\phi\phi}(r) = \frac{R^2}{r^3}\dot{R}$ in the polar and azimuthal directions. The highest shear rate is reached in the radial direction and at the boundary of the bubble, $S_{rr} = -2\frac{\dot{R}}{R}$.

For a cavity of general shape and equivalent radius $R = (\frac{3\Omega}{4\pi})^{1/3}$ growing in an infinite medium while conserving its shape, we consider a thin layer of fluid of thickness $h \ll R$ surrounding the cavity. The volume of this layer of fluid is conserved and can be approximated as $\delta\Omega = CR^2h$ where C is a constant set by the shape of the cavity. If the layer thickness remains spatially uniform during deformation, the shear rate in the radial direction is

$$S_{rr} = \frac{\varepsilon_{rr}}{dt} = \frac{1}{dt}\frac{h(t+dt) - h(t)}{h(t)} = \frac{d \ln h}{dt} = -2\frac{\dot{R}}{R}, \qquad [1]$$

where we use that $h = \delta\Omega C^{-1} R^{-2}$ due to volume conservation.

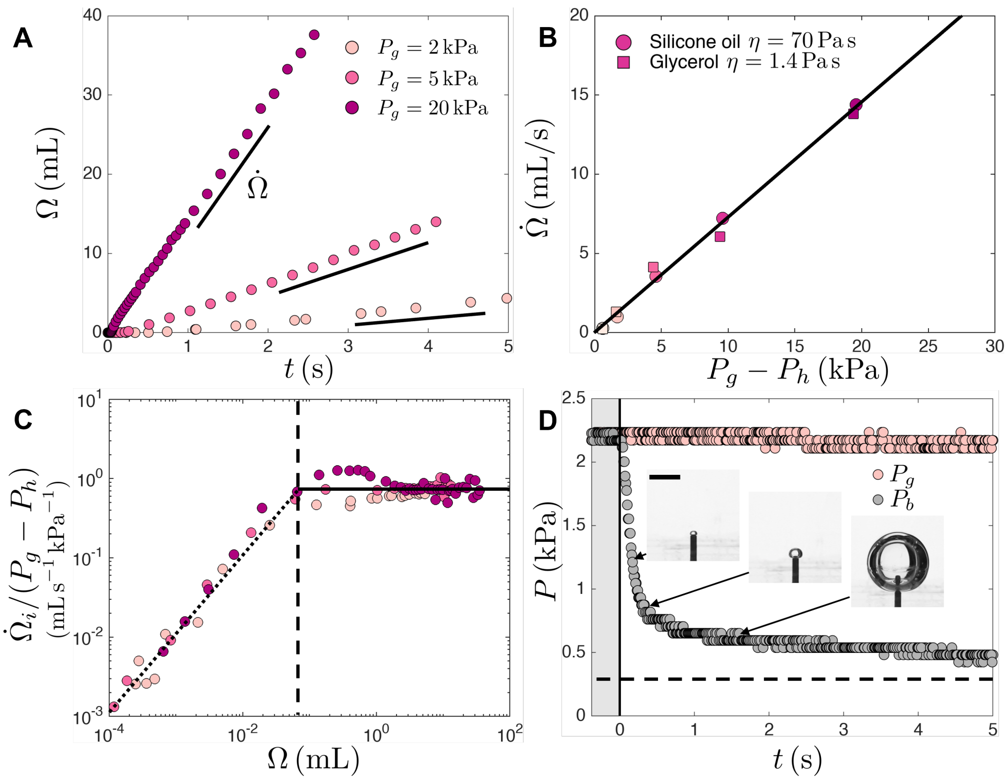

Fig. S1. Growth dynamics of air bubbles in viscous Newtonian liquids. (**A**) Bubble volume Ω measured using image processing *versus* time for air injection into silicone oil with viscosity $\eta = 70\,\mathrm{Pa\,s}$ for different tank gauge pressures P_g. The bubbles grow at a steady-state volume rate $\dot{\Omega}$ during most of the growth. The volume is calculated by assuming radial symmetry. (**B**) The steady-state volume rate $\dot{\Omega}$ increases linearly with $P_g - P_h$ and is the same for bubbles growing in silicone oil ($\eta = 70\,\mathrm{Pa\,s}$) and in glycerol ($\eta = 1.4\,\mathrm{Pa\,s}$), indicating pressure losses in the tubing. The fit yields $\dot{\Omega} = P_g/R_{tubing}$ with the tubing hydraulic resistance $R_{tubing} = 1.38\,\mathrm{mL\,s^{-1}\,kPa^{-1}}$ (black line). (**C**) Instantaneous volume rate $\dot{\Omega}_i$ divided by the pressure difference between the gas tank and the bottom of the container $P_g - P_h$ *versus* bubble volume Ω, revealing two regimes of bubble growth for silicone oil experiments. For volumes smaller than $0.05\,\mathrm{mL}$, the volume rate increases linearly with Ω as $\dot{\Omega}_i/(P_g - P_h) = 3/4\Omega/\eta$ (dotted line). This indicates that the pressure loss at the bubble interface due to the viscous resistance of the liquid dominates over the pressure loss in the tubing. The shear rate $\dot{\Omega}_i/\Omega$ is constant and the bubble volume grows exponentially in time. For volumes larger than $0.05\,\mathrm{mL}$, the volume rate reaches a steady-state value $\dot{\Omega}_i = (P_g - P_h)/R_{tubing}$ (solid line). The viscous resistance of the liquid is negligible and the pressure is lost in the tubing. (**D**) Gauge pressure measured at the exit of the tank (P_g) and at the nozzle inlet (approximately equal to the bubble pressure P_b) for air injection into silicone oil at $P_g = 2.2\,\mathrm{kPa}$. P_g remains constant throughout air injection, while the bubble pressure P_b quickly decreases from $2.2\,\mathrm{kPa}$ to a value close to the hydrostatic pressure P_h (dashed line). This confirms that the tank pressure is predominantly lost in the tubing. The scale bar denotes 2 mm.

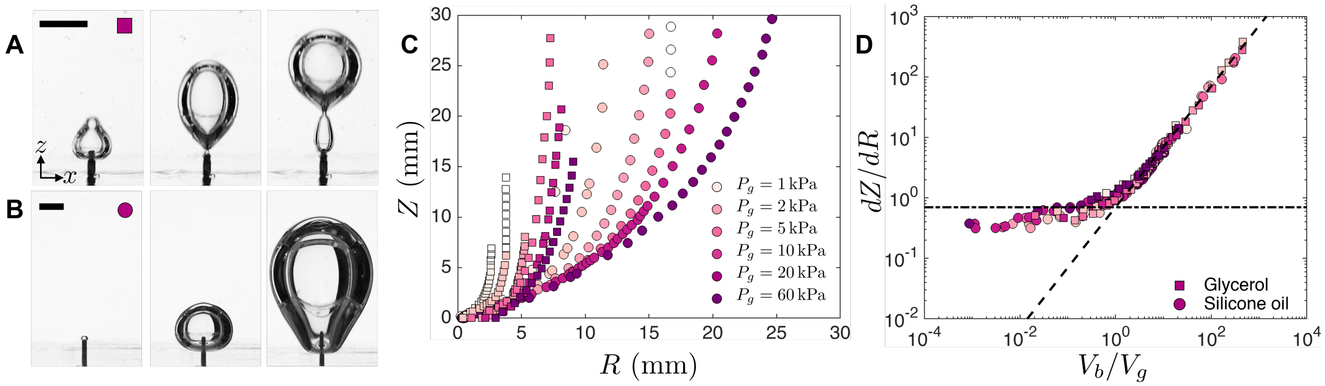

Fig. S2. Vertical motion of air bubbles in viscous Newtonian liquids. (**A**) Images of air injection into glycerol ($\eta = 1.4\,\mathrm{Pa\,s}$) taken at times 0.03 s, 0.10 s, 0.14 s from the beginning of bubble growth. (**B**) Images of air injection into silicone oil ($\eta = 70\,\mathrm{Pa\,s}$) taken at times 0.016 s, 0.16 s, 1.6 s. For both experiments, the gauge pressure is $P_g = 20\,\mathrm{kPa}$ and the scale bar represents 1 cm. (**C**) Z position of the center of the bubble *versus* the equivalent radius $R = (3/(4\pi)\Omega)^{1/3}$. Open symbols indicate bubbles that have detached from the nozzle. (**D**) Rate of change of Z with R *versus* the ratio of the buoyancy velocity and the growth velocity, V_b/V_g. When the growth velocity dominates ($V_b/V_g < 1$), the contribution of buoyancy to the vertical motion is negligible and $\dot{Z} = V_g$ (dash-dot line). When the buoyancy velocity dominates ($V_b/V_g > 1$), the velocity is set by the balance between buoyancy and viscous forces and $\dot{Z} = V_b$ (dashed line).

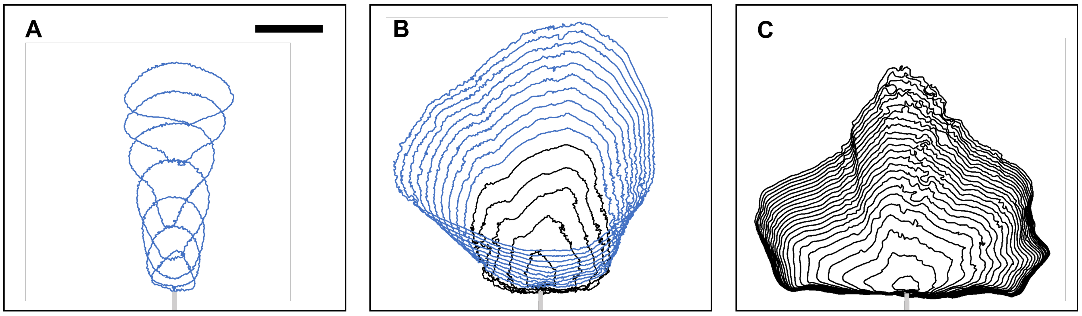

Fig. S3. Growth- and buoyancy-dominated regimes. (**A**) Outline of the air cavity every $1\,\mathrm{s}$ for air injection into a $\phi_m = 0.57$ cornstarch suspension with $P_g = 5\,\mathrm{kPa}$. The bubble rapidly rises and detaches from the nozzle. The scale bar indicates $2\,\mathrm{cm}$. **(B)** Outline of the air cavity every $4\,\mathrm{s}$ for air injection into a $\phi_m = 0.58$ cornstarch suspension with $P_g = 5\,\mathrm{kPa}$. At early times (black contours), the air cavity remains attached to the nozzle as buoyancy effects are negligible. This corresponds to the growth-dominated regime. When the cavity volume becomes large enough, the bottom of the air cavity rises and the cavity detaches from the nozzle (blue contours) as it transitions to the buoyancy-dominated regime. **(C)** Outline of the air cavity every $4\,\mathrm{s}$ for air injection into a $\phi_m = 0.60$ cornstarch suspension with $P_g = 5\,\mathrm{kPa}$. The air cavity remains attached to the nozzle for the entire growth of the bubble as buoyancy effects remain negligible.

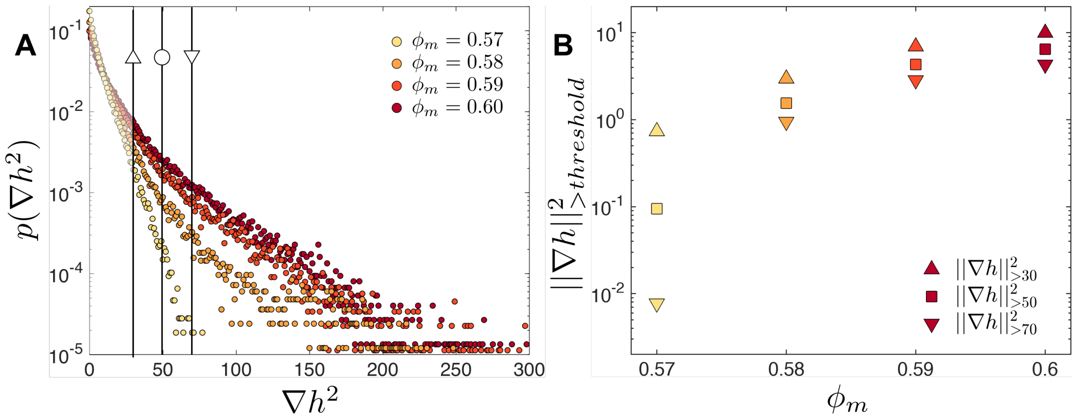

Fig. S4. Dependence of the metric for local fracture characterization $||\nabla h^2||_{>threshold}$ **on the threshold value.** (**A**) Histogram of the air thickness gradient values inside the region of interest for different cornstarch mass fractions ϕ_m. The vertical lines indicate $\nabla h^2 = 30, 50, 70$ which are the threshold values used as lower bounds for the integration in (B). Values of gradient intensity below the threshold value are excluded when calculating the fracture metric. **(B)** Metric for local fracture characterization $||\nabla h||^2_{>threshold} = \int_{threshold}^{\infty} p(\nabla h^2) \mathrm{d}\nabla h^2$ versus cornstarch mass fraction ϕ_m for threshold values of 30, 50 and 70.

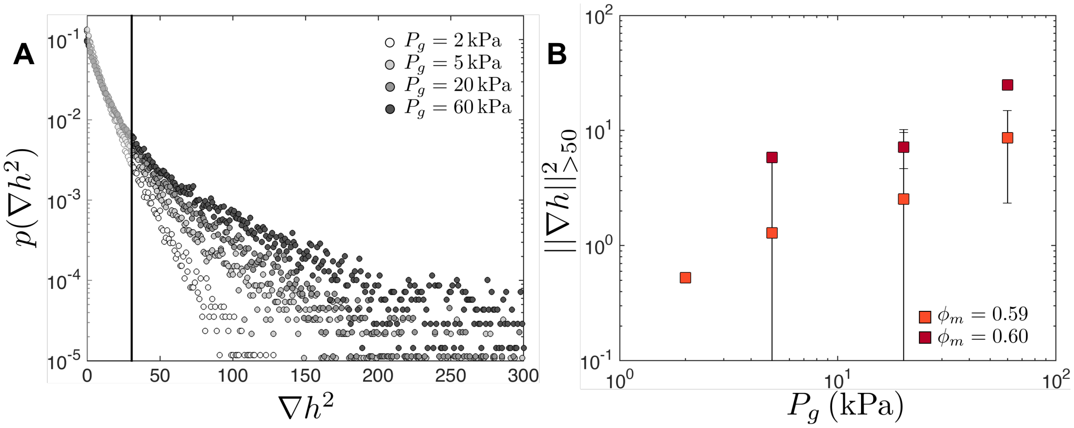

Fig. S5. Dependence of the metric for local fracture characterization $||\nabla h^2||_{>50}$ on the gas tank pressure. (**A**) Histogram of the air thickness gradient values inside the region of interest calculated for $\Omega = 50\,\mathrm{mL}$ at $\phi_m = 0.59$ and for different P_g. **(B)** Metric for local fracture characterization $||\nabla h||^2_{>50} = \int_{50}^{\infty} p(\nabla h^2)\mathrm{d}\nabla h^2$ versus P_g. The metric increases with P_g, indicating that more fractures form. This increase can be related to a larger shear rate $S_{rr} \propto P_g/\Omega$ in the suspension.

Movie S1. Air thickness videos from experiments at cornstarch mass fractions $\phi_m = 0.57$ and 0.60 and tank gauge pressure $P_g = 20\,\mathrm{kPa}$.

Movie S2. Air thickness and air thickness gradient intensity videos from experiments at cornstarch mass fractions $\phi_m = 0.57$ and 0.60 and tank gauge pressure $P_g = 20\,\mathrm{kPa}$.

References

1. EJ Barlow, WE Langlois, Diffusion of Gas from a Liquid into an Expanding Bubble. *IBM J. Res. Dev.* **6**, 329–337 (1962).
